# SLC22 Transporters in the Fly Renal System Regulate Response to Oxidative Stress In Vivo

**DOI:** 10.3390/ijms222413407

**Published:** 2021-12-14

**Authors:** Patrick Zhang, Priti Azad, Darcy C. Engelhart, Gabriel G. Haddad, Sanjay K. Nigam

**Affiliations:** 1 Department of Biology, University of California San Diego, La Jolla, CA 92093, USA; paz005@ucsd.edu (P.Z.); dengelha@ucsd.edu (D.C.E.); 2 Department of Pediatrics, University of California San Diego, La Jolla, CA 92093, USA; pazad@ucsd.edu (P.A.); ghaddad@ucsd.edu (G.G.H.); 3 Department of Neurosciences, University of California San Diego, La Jolla, CA 92093, USA; 4 Rady Children’s Hospital, San Diego, CA 92123, USA; 5 Department of Medicine, University of California San Diego, La Jolla, CA 92093, USA

**Keywords:** organic anion transporter, organic cation transporter, organic zwitterion, remote sensing and signaling theory, drug transporter, acute kidney injury, AKI, OAT1, OAT2, OAT3, OCT1, OCT2, OCT3, URAT1, antioxidant, SLC22A15, SLC22A16, FLIPT1, FLIPT2, OCTN1, OCTN2, CT2

## Abstract

Several SLC22 transporters in the human kidney and other tissues are thought to regulate endogenous small antioxidant molecules such as uric acid, ergothioneine, carnitine, and carnitine derivatives. These transporters include those from the organic anion transporter (OAT), OCTN/OCTN-related, and organic cation transporter (OCT) subgroups. In mammals, it has been difficult to show a clear in vivo role for these transporters during oxidative stress. Ubiquitous knockdowns of related *Drosophila* SLC22s—including transporters homologous to those previously identified by us in mammals such as the “Fly-Like Putative Transporters” FLIPT1 (SLC22A15) and FLIPT2 (SLC22A16)—have shown modest protection against oxidative stress. However, these fly transporters tend to be broadly expressed, and it is unclear if there is an organ in which their expression is critical. Using two tissue-selective knockdown strategies, we were able to demonstrate much greater and longer protection from oxidative stress compared to previous whole fly knockdowns as well as both parent and WT strains (CG6126: *p* < 0.001, CG4630: *p* < 0.01, CG16727: *p* < 0.0001 and CG6006: *p* < 0.01). Expression in the Malpighian tubule and likely other tissues as well (e.g., gut, fat body, nervous system) appear critical for managing oxidative stress. These four *Drosophila* SLC22 genes are similar to human SLC22 transporters (CG6126: SLC22A16, CG16727: SLC22A7, CG4630: SLC22A3, and CG6006: SLC22A1, SLC22A2, SLC22A3, SLC22A6, SLC22A7, SLC22A8, SLC22A11, SLC22A12 (URAT1), SLC22A13, SLC22A14)—many of which are highly expressed in the kidney. Consistent with the Remote Sensing and Signaling Theory, this indicates an important in vivo role in the oxidative stress response for multiple SLC22 transporters within the fly renal system, perhaps through interaction with SLC22 counterparts in non-renal tissues. We also note that many of the human relatives are well-known drug transporters. Our work not only indicates the importance of SLC22 transporters in the fly renal system but also sets the stage for in vivo studies by examining their role in mammalian oxidative stress and organ crosstalk.

## 1. Introduction

Solute carrier proteins (SLCs) are membrane-bound transporters that manage a wide array of substrates such as antioxidants, signaling molecules, metabolites, hormones, nutrients, and neurotransmitters [1]. Through cross-tissue co-expression network analysis, the SLC22 family emerges as a central hub in endogenous metabolite homeostasis—in connection with other SLC and ABC transporters as well as drug metabolizing enzymes (DMEs)—which is consistent with the Remote Sensing and Signaling Theory (RSST) [2,3,4]. Many of these genes are also critical for the absorption, distribution, metabolism and excretion (ADME) of drugs [5].

The connections between SLC22 transporters, other SLC transporters, ABC transporters, and DMEs appear to be particularly important in the regulation of the gut-liver-kidney (GLK) axis where inter-organ crosstalk occurs via transporters and other genes, to mediate signaling, cross-tissue metabolism and ultimately maintain homeostasis, such as after oxidative injury [6,7]. Drug transporters, among the best known of which are multispecific SLC22 transporters such as OAT1 (originally NKT), OAT3, and OCT2, play a vital role in the body’s ability to transport and eliminate drugs [8,9,10,11]. Many compounds often bind to the same transporter, resulting in drug–drug and drug–metabolite interactions. These competing interactions potentially reduce the efficacy and safety of certain pharmaceuticals [12]. These “drug” transporters, however, play essential roles in handling metabolites necessary for organ function and metabolism and therefore their endogenous roles require further study.

While knockout mouse models have been useful for understanding the in vivo biology of SLC22 transporters, the overt phenotypes have for the most part been modest, and only after detailed biochemical and omics analyses, or additional experimental manipulations, have clear endogenous functional abnormalities been revealed [13,14,15,16,17]. *Drosophila melanogaster* (fruit fly) has emerged as a viable model organism for the investigation of ADME genes, due to the similarities between human and fruit fly drug metabolism and excretion physiology [18,19,20]. The fruit fly possesses many tissues and organs such as Malpighian tubules, fat body, and intestinal system which are thought to roughly mirror key aspects of the mammalian kidney, liver, and digestive system, respectively. Moreover, the hindgut, midgut, and crop are sometimes viewed as functionally analogous to the human large intestine, small intestine, and stomach [21]. Due to its ease of use and similarity to mammalian physiology, the fruit fly has been used to investigate a number of disease states such as hypoxia, various cancers, and many metabolic diseases [22,23,24,25,26]. Thus, the fruit fly is potentially a useful organism to explore different aspects of metabolism such as SLC22 transporter modulation of antioxidants and the phenotypic consequences resulting from altered expression.

Previous work has shown that four *D. melanogaster* putative SLC22 orthologs (CG6126, CG4630, CG16727, and CG6006) display a modest increase in resistance to paraquat-induced oxidative stress when knocked down ubiquitously [27]. In this study, tissue specific knockdowns were created and subjected to oxidative stress assays to further elucidate the function of these SLC22s in specific organ systems. Paraquat (PQ) is a herbicide with neurotoxicant characteristics that can induce redox cycling [28]. In such cases, elevated redox cycling leads to an increase in reactive oxygen species (ROSs) which increase oxidative stress within an organism. The four fly SLC22 transporters in question are related to mammalian SLC22s with CG6126, CG16727, and CG4630 being orthologous to SLC22A16 (FLIPT2), A7, and A3, respectively. Additionally, CG6006 is equally orthologous to ten different SLC22 transporter: A1, A2, A3, A6, A7, A8, A11, A12, A13, and A14. Several of the other transporters mentioned above have low level homology to a number of OATs, including SLC22A6 and SLC22A12, both of which are major renal urate and/or antioxidant transporters. Based on the endogenous function of these SLC22 transporters in mammals, it is assumed that disrupting transporter expression in the whole fly has the potential to decrease the excretion of SLC22 antioxidant substrates such as ergothioneine (EGT), carnitine, carnitine derivatives, and uric acid. Decreased excretion presumably results in increased serum levels of endogenous putative antioxidant molecules, which would therefore combat PQ-induced oxidative stress. In mammals, the kidney plays a major role in the elimination of many endogenous antioxidants [7,29]. The implication, therefore, is that the Malpighian tubule might be the critical site of expression of these transporters for protection from oxidative stress. 

To evaluate this hypothesis, we created two sets of tissue-specific RNAi knockdowns using two different tissue specific drivers c42-Gal4 (Driver A) and c591-Gal4 (Driver B). Driver A has been employed to model and investigate the fly renal system [30,31,32]. Driver B expresses knockdown largely in the *Drosophila* equivalent of the “gut-liver-kidney” (GLK) axis—the gut, fat body, and the Malpighian tubule [3,33,34,35,36]. Importantly, one feature common to these two drivers is the ability to drive expression in the Malpighian tubule. 

Thus, if both of the two drivers result in oxidative-stress resistance for one or more of the four aforementioned SLC22 transporters, it supports the view that SLC22 transporter expression in the Malpighian tubule is critical for protection from oxidative stress. As shown, we validate this for several of the SLC22 transporters studied, and the tissue-specific knockdowns were found to provide greater protection than previously reported for the whole fly. The results strongly support the view that SLC22 transporters in the Malpighian tubule are crucial for survival in the face of oxidant stress—though for some transporters, other tissues are likely to also be involved. The study also raises the possibility that the in vivo tissue-specific regulation of oxidative stress response requires one or more fly renal SLC22s, working together with non-renal transporters in the regulation of the redox state [6,37,38].

## 2. Results and Discussion

It has been shown that ADME (administration, distribution, metabolism, and elimination) genes not only handle many drugs, but also many endogenous molecules. These include a broad range of metabolites and signaling molecules [2,39,40]. Tissue co-expression networks support the view that the SLC22 family is central to inter-organ communication, particularly in the gut-liver-kidney (GLK) axis [3,36]. Moreover, SLC22 transporters have been shown to be widely conserved throughout organisms such as fly, mice, and worm [7,17,27,37]. 

### 2.1. Human Relatives of the Four Fly SLC22 Transporters and Their Expression in Malpighian Tubule and Other Tissues

Recently, a reclassification of the SLC22 family of OATs, OCTs, and OCTNs has resulted in their classification into eight subgroups based on sequence, evolutionary history, and functionality [27]. These human subgroups categorize SLC22 transporters into OAT (4 subgroups termed S1–S4), OAT-like, OAT-related, OCTN/OCTN-related, and OCT subgroups. The identification of mammalian relatives of CG6126, CG16727, CG6006, and CG4630 was based on the aforementioned subgroups and DIOPT v9.0 classifications [6,37,38,41]. DIOPT classifies each of these fly transporters as being orthologous to single or multiple human SLC22 transporters, as shown in Table 1. The human ortholog with the greatest weight and rank is observed. The transporters CG6126, CG16727 and CG4630 share homology to mammalian SLC22A16 (FLIPT2), A7 (OAT2), and A3 (OCT3), respectively. Both A16 and A3 have been documented to transport carnitine and its derivatives, while A7 has been associated with uric acid regulation. Furthermore, A16 has been associated with ergothioneine transport [6,42,43,44]. The fourth *Drosophila* transporter CG6006 is deemed equally orthologous to SLC22A1 (OCT1), A2 (OCT2), A3 (OCT3), A6 (OAT1), A7 (OAT2), A8 (OAT3), A11 (OAT4), A12 (URAT1), A13, and A14. Although CG6006 does not have a single mammalian SLC22 counterpart, the 10 associated human SLC22s are all believed to transport some sort of antioxidant-like molecule as shown in Table 1. Although, for the other three fly transporters, the highest-ranking mammalian relative is shown, we note that major renal urate transporters SLC22A6 (OAT1), SLC22A8 (OAT3), and SLC22A12 (URAT1) had a substantial, but lower, rank. Moreover, it is worth noting that these mammalian transporters (e.g., OAT1-3, OCT1-3) are considered among the most important multispecific “drug” transporters in the body.

As described in the Remote Sensing and Signaling Theory, multispecific, oligospecific and monospecific SLC and ABC transporters maintain homeostasis through inter-organ and inter-organismal communication mediated by antioxidants, metabolites, signaling molecules, and gut microbiome products [3,4]. The role of multi-organ axes in mediating organ crosstalk via their transport of endogenous small molecules is a major theme in the theory. For SLC22 transporters, this is supported by recent studies indicating their importance in a gut-liver-kidney remote sensing and signaling network of SLCs, ABCs, and DMEs [3,46,47].

**Figure 1:**
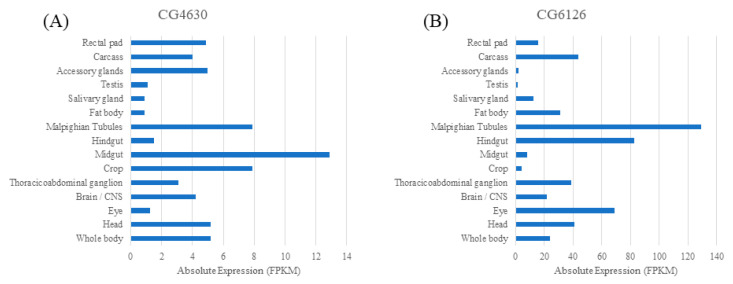

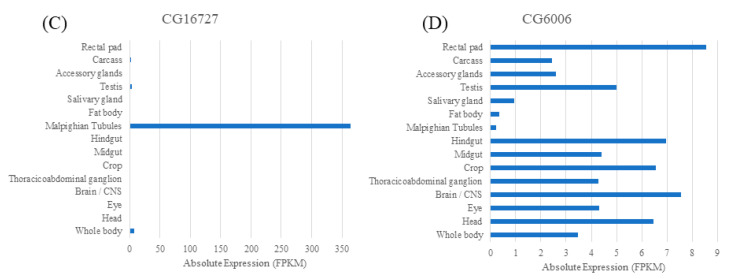
Expression patterns for the four *Drosophila melanogaster* SLC22s CG4630 (**A**), CG6126 (**B**), CG16727 (**C**), and CG6006 (**D**). Created with RNA-seq data procured from Flyatlas2 and measured in FPKM.

Based on expression data shown in Figure 1 and Table 1, it can be said that some of these *Drosophila* transporters may form a putative GLK (gut-fat body-Malpighian tubule) axis. However, the broad expression pattern of these genes (Figure 1) makes it very difficult to determine the importance of expression in a single organ or system. Nevertheless, modest protection from oxidative stress has been demonstrated in non-selective knockdown in the whole fly [27]. The greatest expression per gene can be found in the Malpighian tubule (CG16727 and CG6126), midgut (CG4630), and rectal pad (CG6006) as well as other tissues, including the brain. For 3/4 of these SLC22 genes, the Malpighian tubule is among those tissues that has the greatest expression, which led us to hypothesize a major role of this organ in protection from oxidative stress via SLC22 transporters.

### 2.2. Tissue-Selective Knockdowns

**Figure 2:**
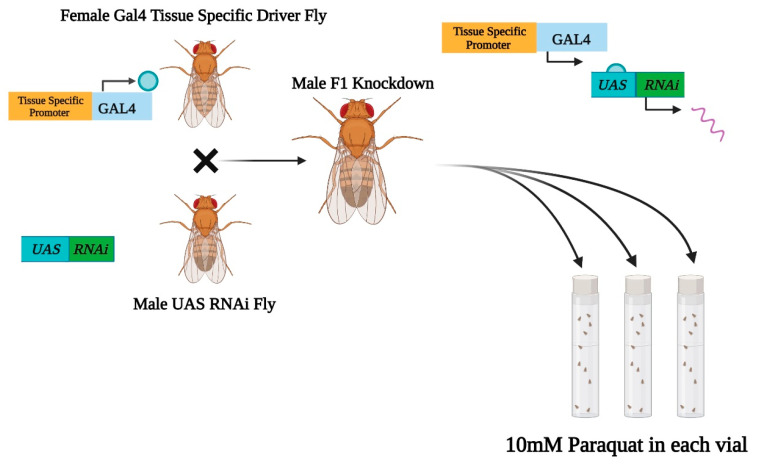
Schematic of the GAL4/UAS (upstream activation sequence) system used to generate RNAi knockdowns. Female Gal4 driver represents the two tissue-selective drivers used being c42-Gal4 (Driver A) and c591-Gal4 (Driver B). Male UAS RNAi fly represent the four BDSC RNAi stocks used (CG6126, CG16727, CG6006, and CG4630).

**Figure 3:**
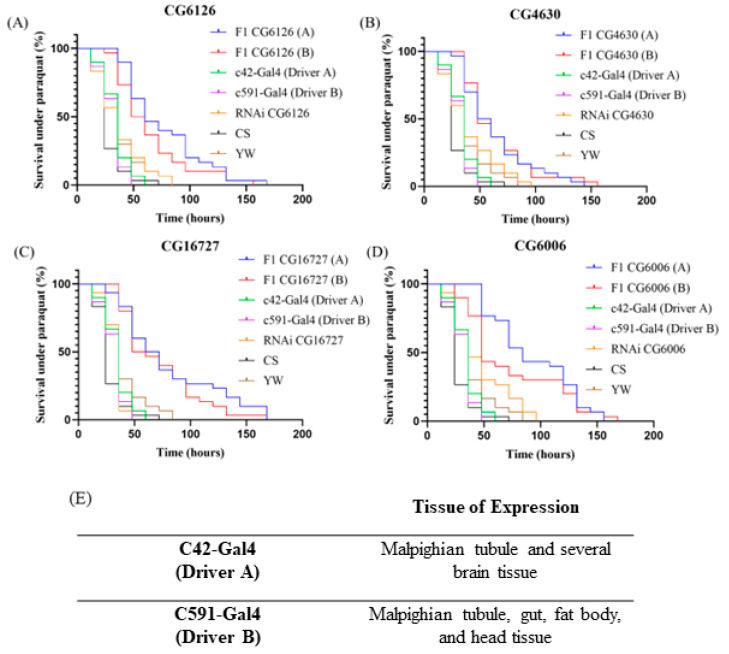
Kaplan-Meier survival curves of tissue specific RNAi knockdowns were created. CG6126 (**A**); CG4630 (**B**); CG16727 (**C**); CG6006 (**D**). All lines were observed for 168 h. Survival was recorded every 12 h. F1 A and B denote RNAi knockdowns created with Drivers A and B, respectively (**E**). RNAi and Gal4 represent parent lines. YW and CS denote wild-type *Drosophila* strains.

To assess the importance of expression in one or a limited set of organs in protection from oxidative stress, tissue specific drivers A and B, shown in Figure 2 and Figure 3E, were used. Both Driver A (c42-Gal4) and Driver B (c591-Gal4) are *Drosophila* lines that express the Gal4 gene in specific tissues, leading to the creation of tissue-selective knockdowns [31,32,33,34]. Driver A allows the expression of RNAi within components of the Malpighian tubule and nervous system [31,32,48,49]. Driver B (c591-Gal4) has been shown to drive expression in the Malpighian tubule and other tissues [35]. Overall, the GAL4/UAS system is a powerful genetic tool that has been utilized in the literature [31,32,33,50]. We used two Gal4 drivers (c42-Gal4 and c591-Gal4) to express our genes of interest in a tissue-specific manner (Figure 2). By testing the oxidative stress resistance of our tissue-specific knockdowns, we were able to further elucidate the functionality of four *Drosophila* SLC22 transporters in specific tissues.

### 2.3. Long Term Survival of Tissue-Selective SLC22 Transporter Knockdowns under Severe Oxidative Stress

Paraquat (PQ) is commonly used on fruit flies to determine if an altered resistance to oxidative stress exists [27,28,51,52,53,54]. In this assay, increased survival indicates a greater resistance to oxidative stress as PQ induces oxidative stress within the flies. Specifically, PQ results in the formation of superoxide radicals, leading to redox cycling that yields a large production of ROSs, resulting in subsequent oxidative stress [55,56,57] (Additional mechanistic insight is provided in 57). Dose-response curves were created for both sets of tissue-selective knockdowns and their respective controls, as shown in Figure 3. F1 lines survived for up to 168 h, and survival was recorded every 12 h. In contrast, a previous analysis of whole fly knockdowns of the same four *D. melanogaster* SLC22 lines resulted in a modest resistance to PQ induced oxidative stress; in that study, it was found that F1 survival ended at 108 h whereas in the present study, using the tissue-specific approach, F1 survived until 168 h [27]. Moreover, in the previous study of whole fly knockdowns, for two of the transporters (CG6006, CG16727), increased survival was seen at only a single point (60H), and for another transporter (CG4630), increased survival was only seen at two time points (36H and 48H). Only CG6126 had an increased survival at three time points (36H, 48H, and 60H); decreased transporter expression resulted in significantly increased resistance (*p* < 0.05) to oxidative stress at these time points, when compared to control lines [27]. In this study, when using tissue-selective drivers, an increased resistance to oxidative stress was observed in as many as nine time points, (*p* < 0.05) when compared to the four controls (Supplementary Figure S4). In addition, overall tissue-specific knockdown survival under oxidative stress lasted up to 168 h whereas control lines (both parent lines, CS WT, YW WT) only survived between 50 and 100 h (Figure 3), demonstrating the greater and longer ability of knockdowns to withstand oxidative stress. 

Figure 3 details the tissue-selective results. It can be seen that the F1 generation of each SLC22 transporter knockdown exhibited increased survival and subsequent resistance to oxidative stress. Log-rank test results (Table 2) show that the overall survival curve of Driver A and B F1 knockdowns are significant (CG6126: *p* < 0.01, CG4630: *p* < 0.01, CG16727: *p* < 0.0001 and CG6006: *p* < 0.01) when compared to the four other control lines (parent and Gal4 driver control lines, wild type yellow white, and wild type Canton S lines). Further statistical analysis shows that there are at least two individual time points for each F1 group, where F1-knockdown survival demonstrates a greater ability to survive under oxidative stress and is statistically significant (CG6126: *p* < 0.05, CG4630: *p* < 0.05, CG16727: *p* < 0.05 and CG6006: *p* < 0.05) when compared to that of the four controls. This information can be found in Supplementary Figures S1–S4. The Driver A knockdown with the greatest number of significant time points was CG6006 (A), with a total of nine (24H, 36H, 48H, 60H, 72H, 84H, 96H, 108H, and 120H). Driver B knockdowns created with CG16727 demonstrated seven statistically significant timepoints (24H, 36H, 48H, 60H, 72H, 84H, and 96H), the greatest number of all Driver B knockdowns. Taken together, there was a marked increase in long term survival of tissue selective SLC22 transporter knockdowns under severe oxidative stress. 

### 2.4. CG6126 (SLC22A16): Potential Involvement in the Transport of Ergothioneine and Carnitine

Regarding CG6126, it is most orthologous to mammalian SLC22A16 (also known as FLIPT2 or CT2), a carnitine and ergothioneine (EGT) transporter. Classification of all SLC22s into subgroups places the closely related transporters A16 (FLIPT2) and A15 (FLIPT1) into the OCTN/OCTN-related subgroup. Previously, the orphaned transporter A15 had no confirmed ligands but both a phylogenetic and transporter assay analysis reveals that A15 shares A16′s affinity for EGT and carnitine, while also transporting carnosine [6,58,59]. Carnitine is believed to possess antioxidant properties including direct ROS scavenging, metal ion chelation, regulation of enzyme generating radicals, defense of mitochondrial integrity, and improvement of antioxidant defense systems [60,61,62]. Figure 1 shows that CG6126 has substantial expression in the Malpighian tubules, gut tissues such as the rectal pad and hindgut, fat body, eye, and carcass. Previously, the PQ assay of the ubiquitous CG6126 yielded three statistically significant time points (36H: *p* < 0.05, 48H: *p* < 0.05, 60H: *p* < 0.05) [27]. However, the PQ assay with the tissue selective Driver A yielded six statistically significant time points (24H: *p* < 0.05, 36H: *p* < 0.05, 48H: *p* < 0.05, 60H: *p* < 0.05, 72H: *p* < 0.05, and 84H: *p* < 0.05), and Driver B yielded four (24H: *p* < 0.05, 48H: *p* < 0.05, 60H: *p* < 0.05, and 84H: *p* < 0.05) (Supplementary Figure S1). For Driver A, a doubling of significant time points was observed compared to ubiquitous knockdowns. Based on its mammalian ortholog, we suggest that CG6126 plays a role in carnitine modulation within a *Drosophila* gut-fat body-Malpighian tubule axis. Our investigation of *Drosophila* SLC22s indicated that another transporter, CG6536, was not only another ortholog of the mammalian carnitine transporter A16, but the knockdown of this gene resulted in lethality of the organism at the pupa stage [27]. Moreover, CG6536 expression was found to be highest within the fly gut-liver-kidney tissue, similar to CG6126. Similarities between CG6126 and CG6536, such as overlapping expression patterns and orthology to mammalian SLC22A16, led us to speculate that the two transporters work together to regulate carnitine within *D. melanogaster*. 

### 2.5. CG16727 (SLC22A7): Potential Regulator of Uric Acid

We found that CG16727 is most orthologous to human SLC22A7 (OAT2) which forms its own SLC22 subgroup OATS2 in the eight subgroup classification of SLC22 [6]. The expression patterns of CG16727 in Figure 1 show large amounts of expression in the Malpighian tubule, with minimal expression in the carcass and testis. Previously, the PQ assay of whole fly knockdown showed only one time point where survival was statistically significant (60H: *p* < 0.05) [27]. In marked contrast, Driver A and Driver B tissue-selective knockdowns both showed significant oxidative stress resistance at seven time points (24H, 36H, 48H, 60H, 72H, 84H, and 132H) and (24H, 36H, 48H, 60H, 72H, 84H, and 96H), respectively (Supplementary Figure S3). In Table 2, survival curves are compared using the log-rank test; compared to the controls, Driver A and Driver B tissue-selective knockdowns showed enhanced survival at a significance level of *p* < 0.0001. Human A7 is known to be expressed within the liver and kidney, where it transports organic anions and zwitterions, including antioxidants such as uric acid [60,63]. In humans specifically, uric acid has been deemed responsible for more than half of all antioxidant activity occurring within serum [63]. By isolating the knockdown to the Malpighian tubule, where CG16727 shows the highest expression by far, and a much greater resistance to oxidative stress was observed compared to the previously published whole-body knockdown. It may be that a decreased elimination of antioxidants by CG16727 in the *Drosophila* Malpighian tubule led to an increased tolerance to ROSs such as hydroxyl radicals and hydrogen peroxide. 

### 2.6. CG6006 (SLC22A1, A2, A3, A6, A7, A8, A11, A12, A13, and A14): Possible Role in Uric Acid Regulation

DIOPT identifies CG6006 as being equally orthologous to human SLC22A1 (OCT1), A2 (OCT2), A3 (OCT3), A6 (OAT1), A7 (OAT2/NLT), A8 (OAT3), A11 (OAT4), A12 (URAT1/Rst), A13, and A14 [41]. Furthermore, SLC22A1, A2, and A3—expressed within the brain, kidney, and liver—fall within the OCT subgroup that has been suggested to have a role in inter-organ communication between the brain and kidney-liver axis. This is drawn from a confirmed ligand profile of monoamine neurotransmitters and other signaling molecules [6]. Both SLC22A6 and A8 fall within the OATS1 subgroup while A7 comprises its own subgroup OATS2. A11 and A12 (URAT1) are members of the OATS3 subgroup. Both A13 and A14 make up the OAT-like subgroup with A13 being known to transport uric acid and A14 having little if any transport data. A common feature between the orthologs found in the OATS1-3 subgroups and SLC22A13 is that they all contribute to the regulation of human uric acid levels, which is known to be a potent antioxidant within humans and flies [6,64]. An altered excretion of antioxidants such as uric acid might create this oxidative-stress resistant phenotype. However, the association of CG6006 and the OCT subgroup, together with high non-renal expression compared to renal expression, suggests a less straightforward interpretation than for the other three transporters studied here, which have high renal expression compared to non-renal expression.

Figure 1 indicates that CG6006 has the greatest expression within the gut tissues, such as the rectal pad, hindgut, and midgut. The rectal pad is a component of the gut system that plays a role in the reabsorption of water and ions, while the hindgut and midgut are traditionally considered the *Drosophila* intestine system [21,65]. It is interesting to note that, despite CG6006 showing modest levels of expression within the Malpighian tubules—while showing the most within gut tissues—both Driver A and Driver B knockdowns produced similar levels of oxidative stress resistance when compared to control lines. Previously, PQ treatment of the ubiquitous CG6006 knockdown showed only a single statistically significant timepoint (60H: *p* < 0.05) [27]. In very marked contrast, Kaplan–Meier curves (Figure 3) of Driver A-specific knockdowns yielded nine significant timepoints where F1 survival under oxidative stress was significant when compared to control lines (24H, 36H, 48H, 60H, 72H, 84H, 96H, 108H, and 120H: *p* < 0.05). Likewise, driver B-specific knockdowns had markedly enhanced survival in the face of oxidative stress (Table 2, Supplementary Figure S4). Driver B affects the expression of Gal4 within not only the *Drosophila* gut-fat body-Malpighian tubule axis but also in the dorsal head and large field neurons, which are both located in the fly’s head. Driver A targets the knockdown into not only the Malpighian tubule, but also the ellipsoid body, pars intercerebralis, fan shaped neurons, and large field neurons which are located in the brain. 

Looking at Figure 1, we see that CG6006 has notable levels of expression within the head and brain. Based on the oxidative stress phenotypes of whole fly (only one significant time point), Driver A (nine significant timepoints), and Driver B (two significant timepoints) knockdowns, we suggest that CG6006, which is related to 10 human SLC22s, is either indirectly or directly involved in the regulation of antioxidants and/or other metabolites within the gut, Malpighian tubule, head, and brain. We cautiously suggest that an inability to handle these antioxidants would lead to systemic accumulation, resulting in an increased protection from oxidative stress. Based on the nine statistically significant time points in the Driver A knockdowns, it can be concluded that CG6006 plays an important role in protection from oxidative stress within the *Drosophila* brain and/or Malpighian tubule. Given the expression patterns (Figure 1) and survival curves with both drivers (Figure 3) for the other three transporters, the importance of expression in the fly renal system is much clearer (with respect to the oxidative stress response). However, in the case of CG6006, it would be useful to, in future studies, separate the brain from the renal contribution with CNS-specific drivers. 

### 2.7. CG4630 (SLC22A3): Potential Role in Handling Carnitine Derivatives 

The fruit fly transporter CG4630 is most orthologous to SLC22A3 (OCT3), a member of the human OCT subgroup that is most expressed in the brain, liver, and kidney [6,41]. Human SLC22A3 regulates monoamine neurotransmitters and carnitine derivatives [6]. Figure 1 expression patterns of CG4630 show greatest expression within the rectal pad, midgut, and Malpighian tubule; the common expression pattern here is the Malpighian tubule, which is the *Drosophila* kidney analog, suggesting CG4630 may have a critical function in the fly renal system. Previous analyses of survival under PQ induced oxidative stress revealed that ubiquitous knockdowns had only two significant time points (36H and 48H: *p* < 0.05) [27]. On the other hand, in this study, the use of tissue-selective knockdowns Driver A yielded four significant time points (24H, 36H, 48H, and 72H: *p* < 0.05) and Driver B yielded two (36H and 72H: *p* < 0.05) (Supplementary Figure S2). Despite having the greatest expression in the midgut and rectal pad, we see that Malpighian tubule-selective knockdowns created with Driver A resulted in the same phenotype as Driver B induced knockdowns. If the function of CG4630 was essential to *Drosophila* gut tissue, it seems likely there would have been a more robust phenotype following the Driver B knockdown since multiple critical tissues would be affected; however, both drivers yielded similar results. Based on this, we suggest that CG4630 may have a role similar to the other three described transporters by regulating potential antioxidants such as carnitine derivatives in the *D. melanogaster* Malpighian tubule.

To summarize, given certain similarities in physiology between *Drosophila* and mammalian models, the fruit fly can be a useful tool in the characterization of understudied transporters. By using Drivers A and B to pinpoint knockdown into specific tissues, we were able to achieve F1 generations of RNAi knockdowns with a much greater resistance to oxidative stress when compared to previous ubiquitous RNAi knockdown results. Although the results strongly support the view that fly renal system SLC22 transporters play a key role in the response to oxidative stress, we note that the tissue-selective drivers we used affected non-renal tissues as well. Having obtained a more robust phenotype, using tissue-selective drivers, the next step might be to evaluate whether levels of antioxidant metabolites are altered in one or more of the four knockdowns. It would also be useful to evaluate changes in gene expression resulting from the knockdown. While the assumption is that the phenotype might be due to altered transport of one or more antioxidants, it is possible that this is not the case for all four of the knockdowns. For example, rather than the altered transport of a specific antioxidant in one or more of the knockdowns, there may be multiple downstream effects on metabolism due to changes in expression of key enzymes or other genes which, collectively, could result in protection from oxidative stress. In other words, while it is attractive to explain the mechanism underlying the phenotype in terms of altered antioxidant transport, multiple possibilities may need to be explored.

Our study sets the stage for a better understanding of the role of the human and murine relatives of these four fly transporters in protection from oxidative stress. The Remote Sensing and Signaling Theory proposes that SLC22 transporters (as well as other “drug” transporter families) work together to promote the homeostasis of endogenous small molecules, by mediating communication between organs and organisms as well as at multiple scales, including cells and organelles [2,3,4]. The theory also describes the way the Remote Sensing and Signaling System/Network resets in response to tissue injury and systemic disruptions. Crucially, oxidative stress acts at multiple scales and in multiple tissues. It has been shown that the SLC22 transporter family is a key hub in the Remote Sensing and Signaling Network, and there is, as discussed, ample in vitro evidence to indicate that mammalian relatives of these SLC22 transporters handle some of the most important antioxidants in the body, including uric acid, ergothioneine, carnosine and carnitine [3,7]. Nevertheless, there is very little in vivo support in humans or other mammals. Our work here, using the fly, suggests that studies in oxidative stress provide a fruitful avenue in the investigation of mammalian SLC22 relatives.

## 3. Materials and Methods

### 3.1. Data Collection

SLC22 human and *Drosophila melanogaster* data were acquired following the data collection procedure published previously [6,27]. Ortholog data was determined using the Drosophila RNAi Screening Center Integrative Ortholog Prediction Tool (DIOPT) version 9.0 produced by the Drosophila RNAi Screening Center (DRSC) [41]. Human genes holding the greatest rank and weight were deemed the most orthologous. 

### 3.2. Drosophila Strains and Genetics:

*Drosophila melanogaster* Gal4 and RNAi lines were purchased from the Bloomington Drosophila Stock Center (Indiana University, Bloomington, IN, USA) [66]. Wild-type Canton-Special and yellow white stocks were obtained from the laboratory of Dr. Gabriel Haddad (University of California San Diego). All flies were fed with a standard cornmeal-molasses-yeast diet and maintained at room temperature. Two tissue specific *GAL4/UAS* drivers c42-Gal4 (BDSC stock #30835) and c591-Gal4 (BDSC stock #30843) were used to downregulate expression of the following lines: CG6126, CG4630, CG16727, and CG6006 in selected tissues as noted in the Results and Discussion. Driver A (c42-Gal4) altered expression in the ellipsoid body, pars intercerebralis, fan shaped neurons, large field neurons, and Malpighian tubule. Driver B (c591-Gal4) altered expression in the trachea, salivary gland, dorsal head, antenna anlagen, gut, fat body, and Malpighian tubule. Male RNAi *Drosophila* SLC22 lines were crossed with virgin female tissue-specific Gal4 drivers to express the downregulation of listed transporters within an F1 generation [67]. *Drosophila* expression data was collected from FlyAtlas2, the *Drosophila* gene expression atlas [68]. FlyAtlas2 expression data is obtained using RNA-seq. Figure 2 was created through https://biorender.com (accessed on 1 November 2021).

### 3.3. Paraquat Exposure of F1 RNAi Flies

All flies were observed throughout their life cycle to account for any unwanted developmental abnormalities. F1 male offspring were aged two to seven days before being exposed to paraquat for resistance to oxidative stress. F1, Gal4 parent, RNAi parent, YW wildtype and CS wildtype were all exposed to PQ simultaneously. Three replicates of each line were tested. Flies were fed on 3 mm Whatmann paper soaked with 10 mM paraquat (*N*,*N*′-dimethyl-4,4′-bipyridinium dichloride, Sigma) (PubChem CID: 15938) in combination with 10% sucrose with fresh PQ added every 24 h. Survival was recorded every 12 h. Flies were never exposed to non-room-temperature conditions. The statistical analysis was performed using GraphPad Prism and Microsoft Excel.

## Figures and Tables

**Table 1:** Subgroup placement of tested *Drosophila* SLC22 transporters, human orthologs, and fly tissue expression. Specific *Drosophila* SLC22 transporters were deemed orthologous to human counterparts based on DIOPT 9.0 and FlyBase classification [45]. Associated metabolites were assigned based on human or mammalian SLC22 functional data. Check mark indicates expression in that tissue. (L) denotes expression level of <1FPKM. n/a: not applicable. (*) listed on A14 denotes orphan transporter status, having no confirmed substrates.

Related SLC22 Human Subgroups	SLC22 Subgroup Members	Drosophila SLC22	SLC22 Human Ortholog	Likely Transported Antioxidants (Mammals)	Drosophila Tissue Expression		
					Gut	Fat Body	Malpighian Tubule
OCTN/OCTN-related	A4, A5, A15, A16, A21	CG6126	A16	Carnitine, carnitine derivatives, and EGT	✓	✓	✓
OATS2	A7	CG16727	A7	Uric acid	n/a	n/a	✓
OCT	A1, A2, A3	CG6006	A1, A2, A3	Carnitine derivatives	✓	✓	✓(L)
OATS1	A6, A8, A20		A6, A8	Uric acid			
OATS2	A7		A7	Uric acid			
OATS3	A11, A12, A22		A11, A12	Uric acid			
OAT-like	A13, A14		A13, A14 *	Uric acid			
OCT	A1, A2, A3	CG4630	A3	Carnitine derivatives	✓	✓(L)	✓

**Table 2:** This table shows *p*-values obtained from log-rank tests when comparing the survival trends depicted in Figure 3. Overall survival curves of the tissue specific knockdowns (F1 of Driver A and B) were compared individually to each control group. This was repeated for each respective control (RNAi parent, driver parent, wild type CS and wild type YW). Both sets of tissue-specific knockdowns show statistically significant differences when individually compared to control groups RNAi, driver, CS, and YW (CG6126: *p* < 0.001, CG4630: *p* < 0.01, CG16727: *p* < 0.0001 and CG6006: *p* < 0.01). The presence of (A) and (B) denotes the driver used to create that respective F1 generation of knockdowns.

	F1 vs. RNAI	F1 vs. Driver	F1 vs. CS	F1 vs. YW
F1 CG6126 (A)	<0.0001	<0.0001	<0.0001	<0.0001
F1 CG4630 (A)	0.0031	<0.0001	<0.0001	0.0003
F1 CG16727 (A)	<0.0001	<0.0001	<0.0001	<0.0001
F1 CG6006 (A)	<0.0001	<0.0001	<0.0001	<0.0001
F1 CG6126 (B)	0.0003	<0.0001	<0.0001	0.0002
F1 CG4630 (B)	0.0023	<0.0001	<0.0001	0.0001
F1 CG16727 (B)	<0.0001	<0.0001	<0.0001	<0.0001
F1 CG6006 (B)	0.0022	<0.0001	<0.0001	<0.0001

## Data Availability

Please see Supplementary Material.

## Supplementary Materials

The following are available online at https://www.mdpi.com/article/10.3390/ijms222413407/s1.

## Abbreviations

SLC22	Solute Carrier Family 22
OAT	Organic Anion Transporter
OCTN	Organic Zwitterion (Carnitine) Transporter
OCT	Organic Cation transporter
FLIPT	Fly-Like Putative Transporter
WT	Wild-Type
URAT	Urate Transporter
CT	Carnitine Transporter
DME	Drug Metabolizing Enzyme
RSST	Remote Sensing and Signaling Theory
ADME	Administration, Distribution, Metabolism, and Excretion
GLK	Gut-Liver-Kidney
NKT	Novel Kidney Transporter
ROS	Reactive Oxygen Species
PQ	Paraquat
UAS	Upstream Activation Sequence
RNAi	Interfering RNA
DIOPT	Drosophila RNAi Screening Center Integrative Ortholog Prediction Tool
ABC	ATP-Binding Cassette
BDSC	Bloomington Drosophila Stock Center
YW	Yellow White
CS	Canton S
EGT	Ergothioneine
