# Supplementary material for: SLC22 Transporters in the Fly Renal System Regulate Response to Oxidative Stress In Vivo
Source: Int J Mol Sci. 2021 Dec 14;22(24):13407. doi: 10.3390/ijms222413407 (PMC8706193; doi:10.3390/ijms222413407)
Supplement: Supplementary file 1 [file ijms-22-13407-s001.zip › ijms-1484415-supplementary.pdf]

(A)

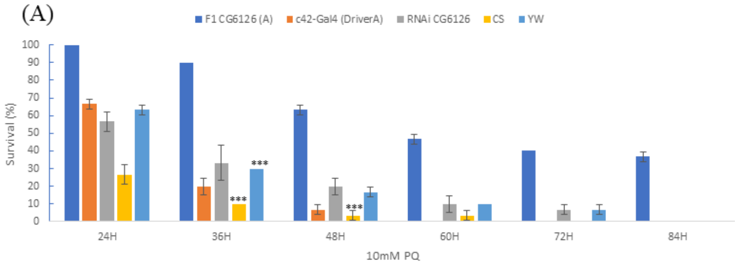

	F1 v c42-Gal4 (DriverA)	F1 v RNAi	F1 v CS	F1 vs YW
24 hours	0.0098525	0.0228602	0.0081634	0.0081634
36 hours	0.0067341	0.0421585	***	***
48 hours	0.0034424	0.0058652	***	0.0198039
60 hours	0.0050633	0.0081634	0.0058652	0.0081634
72 hours	***	0.0098525	***	0.0098525
84 hours	0.0081634	0.0081634	0.0081634	0.0081634

(B)

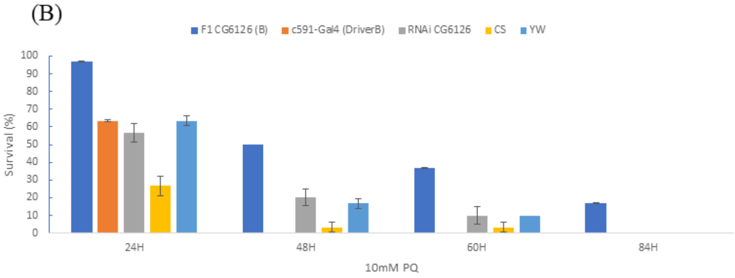

	F1 v c591-Gal4 (DriverB)	F1 v RNAi	F1 v CS	F1 vs YW
24 hours	0.0098525	0.0202041	0.0067341	0.0098525
48 hours	***	0.0350987	0.0050633	0.0098525
60 hours	0.0081634	0.0152681	0.0098525	0.0152681
84 hours	0.0377496	0.0377496	0.0377496	0.0377496

Supplementary Figure S1: Knockdowns generated with CG6126 and Driver A and B, respectively, show resistance to paraquat induced oxidative stress. CG6126 Driver A knockdowns were created using parent lines: BDSC Stock 56038 (CG6126) and BDSC Stock 30835 (driver A). Each time point listed has shown statistical significance ($p < 0.05$) when total survival was compared at that moment, unless noted otherwise. Error bars represent standard error. *** is used to indicate time points where statistical analysis via T-test yielded error due to the nature of the data. However, based on other significant timepoints, we expect these indicated points to show increased resistance as well. Data in graph A of Supplementary Figure 1 show comparisons between knockdowns generated with BDSC Stock 56038 (CG6126) and BDSC Stock 30835 (Driver A). Data in graph B of Supplementary Figure 1 show comparisons between knockdowns generated with BDSC Stock 56038 (CG6126) and BDSC Stock 30843 (Driver B). Alongside graphs A and B are tables that display associated p-values.

(A)

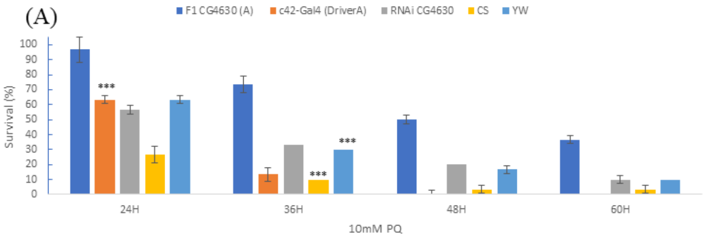

	F1 v c42-Gal4 (DriverA)	F1 v RNAi	F1 v CS	F1 vs YW
24 hours	***	0.0315040	0.0198039	0.0098525
36 hours	0.0130725	0.0377496	***	***
48 hours	0.0058652	0.0198039	0.0050633	0.0098525
60 hours	0.0081634	0.0741799	0.0377496	0.0152681

(B)

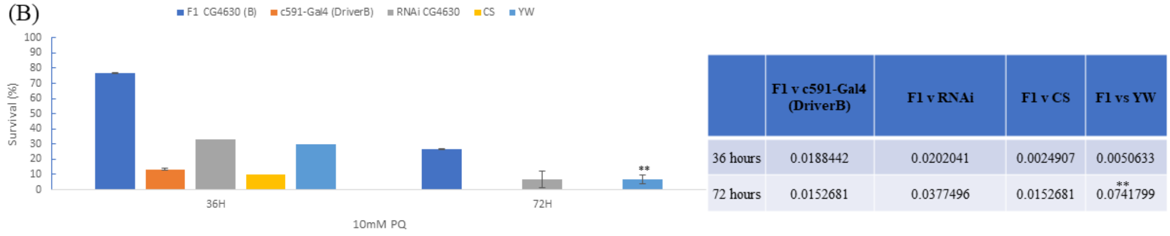

	F1 v c591-Gal4 (DriverB)	F1 v RNAi	F1 v CS	F1 vs YW
36 hours	0.0188442	0.0202041	0.0024907	0.0050633
72 hours	0.0152681	0.0377496	0.0152681	** 0.0741799

Supplementary Figure S2: Knockdowns generated with CG4630 and Driver A and B, respectively, show resistance to paraquat induced oxidative stress. CG4630 driver A knockdowns were created using parent lines: BDSC Stock 61249 (CG4630) and BDSC Stock 30835 (driver A). Each time point listed has shown statistical significance ($p < 0.05$) when total survival was compared at that moment, unless noted otherwise. Error bars represent standard error. *** is used to indicate time points where statistical analysis via T-test yielded error due to the nature of the data. However, based on other significant timepoints, we expect these indicated points to show increased resistance as well. ** indicates p-value > 0.05 for that specific comparison and timepoint. Data in graph A of Supplementary Figure 2 show comparisons between knockdowns generated with BDSC Stock 61249 (CG4630) and BDSC Stock 30835 (Driver A). Data in graph B of Supplementary Figure 2 show comparisons between knockdowns generated with BDSC Stock 61249 (CG4630) and BDSC Stock 30843 (Driver B). Alongside graphs A and B are tables that display associated p-values.

(A)

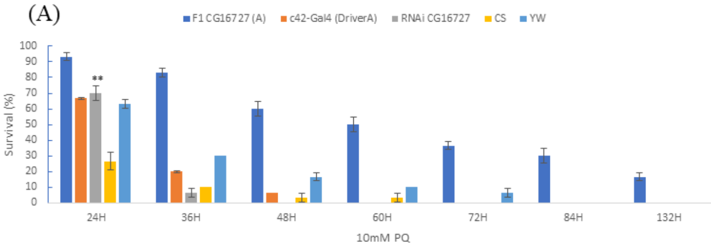

	F1 V C42-GAL4 (DRIVERA)	F1 V RNAI	F1 V CS	F1 VS YW
24 hours	0.0152681	0.0728274 **	0.0170536	0.0000000 *
36 hours	0.0188442	0.0018850	0.0020597	0.0038835
48 hours	0.0152681	0.0091326	0.0233755	0.0058652
60 hours	0.0130725	0.0130725	0.0339082	0.0202041
72 hours	0.0081634	0.0081634	0.0081634	0.0350987
84 hours	0.0350987	0.0350987	0.0350987	0.0350987
132 hours	0.0377496	0.0377496	0.0377496	0.0377496

(B)

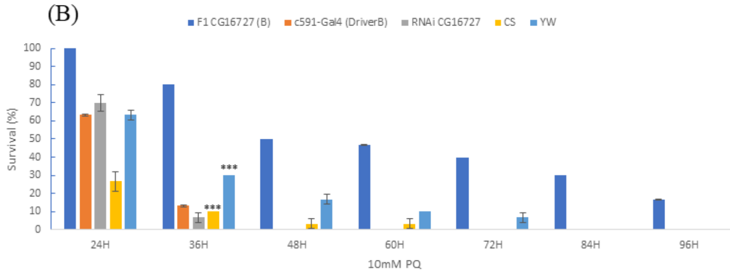

	F1 v c591-Gal4 (DriverB)	F1 v RNAi	F1 v CS	F1 vs YW
24 hours	0.0315040	0.0350987	0.0081634	0.0081634
36 hours	0.0098525	0.0020597	***	***
48 hours	***	***	0.0050633	0.0098525
60 hours	0.0050633	0.0050633	0.0058652	0.0081634
72 hours	***	***	***	0.0098525
84 hours	***	***	***	***
96 hours	0.0377496	0.0377496	0.0377496	0.0377496

Supplementary Figure S3: Knockdowns generated with CG16727 and Driver A and B, respectively, show resistance to paraquat induced oxidative stress. CG16727 driver A knockdowns were created using parent lines: BDSC Stock 57434 (CG16727) and BDSC Stock 30835 (driver A). Each time point listed has shown statistical significance ($p < 0.05$) when total survival was compared at that moment, unless noted otherwise. Error bars represent standard error. *** is used to indicate time points where statistical analysis via T-test yielded error due to the nature of the data. However, based on other significant timepoints, we expect these indicated points to show increased resistance as well. ** indicates p-value > 0.05 for that specific comparison and timepoint. Data in graph A of Supplementary Figure 3 show comparisons between knockdowns generated with BDSC Stock 57434 (CG16727) and BDSC Stock 30835 (Driver A). Data in graph B of Supplementary Figure 3 show comparisons between knockdowns generated with BDSC Stock 57434 (CG16727) and BDSC Stock 30843 (driver B). Alongside graphs A and B are tables that display associated p-values. * indicates a p-value of 4.1119371E-18 for F1 CG16727 (A) vs YW.

(A)

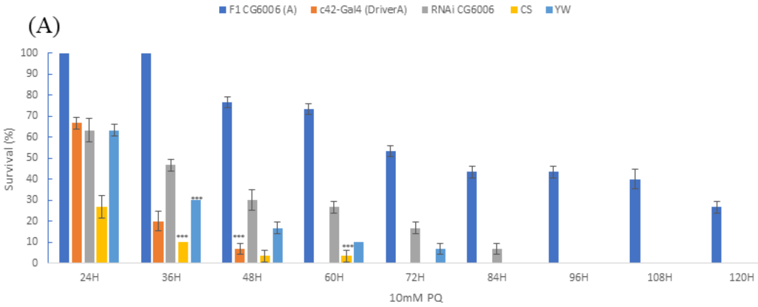

	F1 v c42-Gal4 (DriverA)	F1 v RNAi	F1 v CS	F1 vs YW
24 hours	0.0098525	0.0315040	0.0081634	0.0081634
36 hours	0.0051680	0.0038835	***	***
48 hours	***	0.0198039	0.0020597	0.0091326
60 hours	0.0020597	0.0050633	***	0.0027586
72 hours	0.0038835	0.0081634	0.0038835	0.0198039
84 hours	0.0058652	0.0081634	0.0058652	0.0058652
96 hours	0.0058652	0.0058652	0.0058652	0.0058652
108 hours	0.0202041	0.0202041	0.0202041	0.0202041
120 hours	0.0152681	0.0152681	0.0152681	0.0152681

(B)

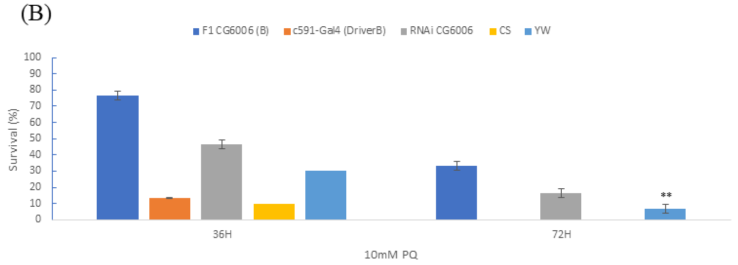

	F1 v c591-Gal4 (DriverB)	F1 v RNAi	F1 v CS	F1 vs YW
36 hours	0.0027586	0.0350987	0.0024907	0.0050633
72 hours	0.0098525	0.0377496	0.0098525	** 0.0571910

Supplementary Figure S4: Knockdowns generated with CG6006 and Driver A and B, respectively, show resistance to paraquat induced oxidative stress. CG6006 Driver A knockdowns were created using parent lines: BDSC Stock 55282 (CG6006) and BDSC Stock 30835 (Driver A). Each time point listed has shown statistical significance ($p < 0.05$) when total survival was compared at that moment, unless noted otherwise. Error bars represent standard error. *** is used to indicate time points where statistical analysis via T-test yielded error due to the nature of the data. However, based on other significant timepoints, we expect these indicated points to show increased resistance as well. ** indicates $p > 0.05$ for that specific comparison and timepoint. Data in graph A of Supplementary Figure 4 show comparisons between knockdowns generated with BDSC Stock 55282 (CG6006) and BDSC Stock 30835 (Driver A). Data in graph B of Supplementary Figure 4 show comparisons between knockdowns generated with BDSC Stock 55282 (CG6006) and BDSC Stock 30843 (Driver B). Alongside graphs A and B are tables that display associated p-values.
